# Comparison of olfactory function, cognitive function and serum tumor necrosis factor-α between bipolar and schizophrenic patients in the remission stage

**DOI:** 10.1186/s12888-023-05330-6

**Published:** 2023-11-07

**Authors:** Langjun Su, Xianlin Liu, Yingying Li, Huiqian Yuan, Qiping Li, Chunyang Li

**Affiliations:** Department of Psychiatry, Shunde WuZhongpei Memorial Hospital, No. 7 Baolin Road, Daliang Town, Shunde District, Foshan City, 528300 Guangdong P.R. China

**Keywords:** Bipolar disorder, Schizophrenia, Remission stage, Olfactory sensitivity, Olfactory identification, Cognitive function, Tumor necrosis factor-α

## Abstract

**Objectives:**

Olfactory function, serum tumor necrosis factor-α (TNF-α) and cognitive function were compared between bipolar disorder (BD) and schizophrenia (SP) patients in the remission stage combined with correlation analysis, with the aim of identifying new indicators for the auxiliary diagnosis of these psychiatric illnesses.

**Methods:**

A total of 46 euthymic BD patients, 42 clinically stable SP patients and 42 healthy controls (HC) were included in this study. Olfactory sensitivity (OS) and olfactory identification (OI) were assessed using Sniffin’ Sticks test, and serum TNF-α levels were measured by ELISA. Clinical symptoms were evaluated with the Hamilton Rating Scale for Depression, Young Mania Rating Scale, Hamilton anxiety scale, and the Positive and Negative Syndrome Scale (PANSS). Social function was evaluated with the Global Assessment Function (GAF) scale. Cognitive function was evaluated using the Trail Making Test-A (TMT-A) and Digit Cancellation Test (DCT).

**Results:**

OI and cognitive function scores and serum TNF-α levels were significantly lower in the BD and SP patients compared with the HC participants. There was no significant difference between the BD and SP groups, and there were no significant differences in OS among the three groups. OI score was positively correlated with years of education in both the BD and SP groups. OI score in the SP group was negatively correlated with age and PANSS score, and positively correlated with GAF score. In the BD group, OS was positively correlated with DCT II and DCT III. In the SP group, OS and OI scores were positively correlated with DCT III, and negatively correlated with TMT-A time. Furthermore, there was a positive correlation between TNF-α and DCT II in the BD group. There was no significant linear correlation between olfactory function and TNF-α in the BD or SP group.

**Conclusion:**

OI may be a trait marker for BD and SP. Some cognitive functions may be correlated not only with TNF-α in BD patients in remission, but also with olfactory function in BD and SP patients in remission.

## Introduction

Studies have shown that patients with BD and SP have olfactory dysfunction [1]. Compared with healthy people, patients with BD and SP have functional impairment in olfactory sensitivity (OS) and olfactory identification (OI), with SP patients showing greater impairment [2]. Imaging studies have revealed anatomical and functional abnormalities in the anterior cingulate cortex, striatum, amygdala, ventromedial prefrontal cortex and orbitofrontal lobe in patients with BD [3–6], while abnormal brain areas in patients with SP include the amygdala, anterior cingulate gyrus, olfactory bulb, orbitofrontal lobe, hippocampus and internal capsule [7, 8]. These areas play an important role in olfactory processing. However, studies on olfactory function in BD and SP patients in the remission stage are limited, and the results have been inconsistent. Some studies have shown that BD patients in remission have defects in olfactory function. Li et al. [2] evaluated olfactory function in 35 patients with BD in remission, and found a deficit in OI compared with HCs, but found no difference in OS between the two groups. However, Kazour et al. [9] reported no significant difference in OS or OI between patients with BD in remission and HCs. Furthermore, while OI function was reported to be significantly lower in SP patients in remission compared with HCs [10], another study showed that, compared with the HC group, OI and OS in SP patients in remission were not significantly different [11].

Studies directly comparing olfactory function between SP and BD patients are limited. Cumming et al. [12] found that OI impairment in the BD group was less than that in the SP group. The study included patients in remission, as well as a combination of patients in the episodic and remission stages. However, no study has directly compared olfactory function between BD and SP patients in remission.

Impairment of cognitive function has been reported in patients with BD and SP [13–15]. Most patients are considered to be in the acute phase, with significant cognitive impairment. However, during remission, even if clinical symptoms stabilize and cognitive function improves to an extent, there may still be impairment. Sánchez-Morla et al. reported that patients with BD in remission have extensive neurocognitive deficits, affecting all cognitive domains such as executive function, attention, speech and visual memory, similar to patients with SP. Persistent speech memory impairment in patients with BD is clinically significant because it is associated with poor psychosocial functioning [16].

Studies have shown that BD and SP may share a common pathophysiology, namely, a mild systemic inflammation/immune disorder-mediated mechanism [17].

Reports on TNF-α in BD and SP patients in remission have been inconsistent. Some studies showed no statistically significant difference in serum TNF-α levels between BD patients in remission and HCs [18, 19]. However, Pietruczuk et al. [20] reported that serum TNF-α levels were significantly lower in BD II patients in remission compared with HCs. Moreover, while some studies found no difference in serum TNF-α between SP patients in remission and HCs [21, 22], Maes et al. [23] reported that serum TNF-α was significantly higher in SP patients in remission compared with HCs.

At present, there is no study directly comparing olfactory function between BD and SP patients in remission, nor has any correlation analysis been performed for olfactory function, cognitive function, and serum TNF-α in these patients. Therefore, in the present study, we compared olfactory function in patients with BD and SP in remission with HCs, with the aim of evaluating whether olfactory function could be used as an aid in the diagnosis or differential diagnosis of these disorders. We performed correlation analysis for olfactory function and cognitive function, as well as serum TNF-α level, among the groups. Our findings should provide a clinical basis for testing new hypotheses on the pathophysiological mechanisms of BD and SP.

## Methods

### Participants

A total of 46 euthymic BD patients and 42 clinically stable SP patients were recruited from July, 2022 to November, 2022 at Shunde WuZhongpei Memorial Hospital in Guangdong, China. A total of 46 healthy hospital staff with social demographic characteristics matched with the patient group were selected as the HC group. The demographic characteristics of our study samples are all Han Chinese.

All patients were diagnosed by trained psychiatrists according to the DSM-5. The patients were between 18 and 60 years of age, with at least primary school education. Clinical symptoms were evaluated with the Hamilton Rating Scale for Depression (HAMD), the Hamilton Rating Scale for Anxiety (HAMA) and Young Mania Rating Scale (YMRS). Euthymic BD patients scored ⩽7 on these scales. Clinically stable SP patients scored < 50 on the Positive and Negative Syndrome Scale (PANSS). All patients were free of upper respiratory tract infections, nasal diseases (including flu like syndrome), traumatic head injuries, other serious illness and drug abuse or alcohol addiction, and all had an IQ ⩾80. The inclusion criteria for control participants were good physical health and no history of mental disorders, neurologic diseases, or drug abuse.

The study was approved by the Medical Ethics Committee of Shunde Wu Zhongpei Memorial Hospital. All participants signed written informed consent.

### Clinical evaluation

A self-designed questionnaire was used to collect basic data, including age, gender, years of education, smoking, and physical health. For the patient groups, clinical data, such as diagnosis, clinical classification, duration of illness, family history, and medication were recorded.

The HAMD scale has been widely used in the clinical evaluation of depression. In this study, the 24-item version (HAMD-24) was used. The total score ranges from 0 to 76, with a score of less than 8 being considered symptom-free. A score of 20 to 35 indicates mild to moderate depressive symptoms. A score greater than 35 indicates symptoms of major depression.

The HAMA scale is widely used to assess the severity of anxiety symptoms in participants. The scale consists of 14 items, with scores ranging from 0 to 56 points. A total score less than 6 indicates no anxiety, 14 points or more indicates definite anxiety, and 21 points or more indicates significant anxiety.

The YMRS scale is widely used to evaluate manic symptoms in the clinic. The scale consists of 11 items, with scores ranging from 0 to 56. Seven items were scored from 0 to 4 points on the severity scale, and the remaining four items were scored from 0 to 8 points. Scores of 0–5 indicate no obvious manic symptoms, 6–10 points indicate manic symptoms, and 22 points indicate severe manic symptoms.

The PANSS scale is mainly used to assess the severity of clinical symptoms in SP. The 30 items in the scale were scored from 1 to 7 points, according to the severity of symptoms (mild to severe), with a total score of 30–210 points.

The Global Assessment Function (GAF) scale is divided into 10 grades, from 1 to 10, with a total score ranging from 0 to 100. It is widely used in the evaluation of psychological, social, and professional functions. The higher the score, the better the social function and the milder the illness.

Each scale was assessed in a quiet environment. HAMD, HAMA, PANSS, and YMRS were assessed based on the subject’s condition in the last week, and GAF was assessed based on the patient’s condition in the last month. The scale was evaluated by two trained psychiatrists.

### Olfactory function

We used the Sniffin’ Sticks test (SST) to assess OI and OS. The OI test consists of 16 olfactory sticks representing different odors. After smelling each stick, the participants choose the one they think is right among the four odor options. If the scent is correctly identified, it is scored as 1 point, with a total score of 0–16. The higher the OI score, the better the OI ability. OS was evaluated using 16 different dilution levels of *n*-butanol as the olfactory agent. Each group was tested with one olfactory stick containing n-butanol and two blank control sticks. Participants were asked to identify the stick containing *n*-butanol from three sticks. OS was measured using the single-staircase method—when the participants correctly identified *n*-butanol twice or failed to identify the odor at the same concentration, the score was recorded and the order of testing was reversed. A higher OS score indicates better OS [24]. All participants were performed the assessment after meal.

### Cognitive testing

TMT-A was used to evaluate processing speed, attention, and cognitive ordering ability. The subject is required to connect the digital circles from 1 to 25 randomly distributed on the paper at the fastest speed in the order from smallest to largest within a time of 300 s.

The DCT task is divided into three parts. In the first part, DCT I, the participants are required to cross out all “3”s from the 25 × 40 number table. This test is mainly used to evaluate attention span and stability. The second part, DCT II, in which the participants need to cross out all the numbers that are not “7” in front of “3” in the number table, is mainly used to evaluate the attention transfer. The third part, DCT III, in which participants are required to cross out all the number “7”s immediately before “3” in the number table, is mainly used to evaluate the distribution of attention. Each part is limited to 3 min, and the unfinished number is recorded as the missing number. Calculation method: Net score = correct number − (number of mistakes + 1/2 missed number).

### TNF-α

A 5-mL volume of fasting venous blood was extracted in the morning, coagulated at room temperature for 20 min, centrifuged at 3,000 r/min for 10 min, and serum was separated. After centrifugation, the upper serum was collected and stored in the refrigerator at − 40 °C for analysis. Serum TNF-α levels were detected by enzyme-linked immunosorbent assay (ELISA). The procedures were strictly according to the instructions attached to the kit.

### Statistical analysis

SPSS 25.0 statistical software was used for statistical analysis. First, the measurement data were tested for normality and homogeneity of variance. Normally distributed data were expressed as mean ± standard deviation, while non-normally distributed data were expressed as median (lower quartile, upper quartile) [M (QL, QU)]. The comparison between the two means was conducted by independent sample *t*-test, paired sample *t*-test or non-parametric test. The mean of multiple groups was compared, and one-way analysis of variance was used for normally distributed data. Non-parametric test (Kruskal–Wallis *H*-test) was used to compare the mean of non-normal data between groups. If there was a statistical difference, pairwise comparison was further conducted, and the Bonferroni method was used to correct the *P*-value. Count data were expressed as the number of cases and component ratio, and the Chi-square test was used for comparison between groups. Pearson correlation analysis (for normally distributed data) or Spearman correlation analysis (for non-normally distributed data) was used according to the data distribution type. The test level was *α* = 0.05, and the two-sided test was conducted.

## Results

### Clinical characteristics

Table 1 summarizes the demographic and clinical information of euthymic BD patients (*n* = 46), clinically stable SP patients (*n* = 42) and control participants (*n* = 46). There were no significant differences in age, gender, smoking status or years of education among the groups (*P* > 0.05), and there was no significant difference in disease course between the BD group and the SP group (*P* > 0.05).

**Table 1:** Clinical characteristics, olfactory function, cognitive function and TNF-α among all groups

Variable	BD group (N = 46)	SP group (N = 42)	HC group (N = 46)	*χ* ^*2*^ */H*	P Value
**Sociodemographics**					
Sex (M/F)	25/21	28/14	20/26	4.761	0.093
Age (years)	37.4 ± 10.6	40.9 ± 9.5	39.9 ± 8.1	1.588	0.208
Education (years)	12 (9, 15)	11.5 (8, 13)	13.5 (8.8, 15)	5.172	0.075
Smoking, N (%)	13 (15.2)	12 (28.6)	7 (15.2)	2.893	0.235
Duration of illness (years)	13.6 ± 8.2	14.9 ± 9.1		0.508	0.478
**Clinical characteristics**					
HAMD	3 (2, 5)		1 (0, 4)		
YMRS	3 (1, 5)		0 (0, 2)		
HAMA	2 (1, 4)		1 (0, 3)		
PANSS		42 (37, 48)	30 (30, 31)	66.34	< 0.001*
GAF	81 (73.8, 86.5)	79 (69.3, 88)	95 (93, 96)	76.815	< 0.001*
**Medications**					
Atypical antipsychotics, N (%)	45 (97.8)	40 (95.2)		0.447	0.504
Risperidone	14 (30.4)	14 (33.3)		0.085	0.771
Olanzapine	9 (19.6)	10 (23.8)		0.234	0.629
Quetiapine	15 (32.6)	3 (7.1)		8.751	0.003*
Mood stabilizers, N (%)	44 (95.7)	21 (50.0)		23.701	< 0.001*
Benzodiazepines, N (%)	16 (34.8)	13 (31.0)		0.146	0.703
**Olfactory function**					
OS	7 (5.9, 10)	7 (5.5, 8.5)	7.5 (5.5, 8.5)	0.561	0.755
OI	12 (9, 14)	11 (10, 13)	14 (12.8, 15)	25.419	< 0.001*
**Cognitive function**					
TMT-A time (seconds)	51 (39.7, 67.3)	54.5 (41, 74.3)	40.5 (29, 53.5)	15.897	< 0.001*
DCT score I	37.5 (32.6, 42)	37.5 (25.1, 42.4)	42 (37.5, 43.5)	9.508	0.009*
DCT score II	35.3 (24, 40.5)	32.3 (20.3, 39)	40.3 (34.5,40.9)	14.671	0.001*
DCT score III	12.8 (8.4, 17.5)	14.5 (8.5, 17.5)	17.5 (14.5, 19)	14.761	0.001*
**TNF-α (pg/ml)**	16.2 (14.3, 18.6)	17.5 (13.8, 19.9)	22.2 (20.9, 23.8)	23.717	< 0.001*

BD: bipolar disorder; SP: schizophrenia; HC: healthy controls; HAMD: Hamilton Rating Scale for Depression; YMRS: Young Mania Rating Scale; HAMA: Hamilton Rating Scale for Anxiety; PANSS: Positive and Negative Syndrome Scale; M/F: male/female; GAF: Global Assessment Function; OS: olfactory sensitivity; OI: olfactory identification; TMT-A: Trail Making Test-A; DCT: Digit Cancellation Test; TNF-α: tumor necrosis factor-α

*P < 0.05

### Comparison of cognitive function among the three groups

As shown in Table 1, cognitive function (including TMT-A, DCT I, DCT II and DCT III) in the BD, SP and HC groups significantly differed (*P* < 0.05). Kruskal–Wallis *H*-test was further used to compare cognitive function among the groups, and the Bonferroni method was used to correct the *P*-value. Cognitive function was significantly decreased in BD and SP patients in remission compared with the HC group. There was no significant difference in cognitive function between BD patients in remission and SP patients in remission (Table 2).

**Table 2:** Pairwise comparison of OI, cognitive function and TNF-α among the groups

Variable	BD group vs. HC group		SP group vs. HC group		BD group vs. SP group	
	H	P Value	H	P Value	H	P Value
OI	3.868	< 0.001*	4.711	< 0.001*	0.931	1.000
TMT-A time	-2.933	0.010*	-3.789	< 0.001*	−0.924	1.000
DCT score I	2.494	0.038*	2.800	0.015*	0.363	1.000
DCT score II	3.134	0.005*	3.453	0.002*	0.391	1.000
DCT score III	3.478	0.002*	3.123	0.005*	−0.276	1.000
TNF-α	-4.627	< 0.001*	-3.587	0.001*	−0.933	1.000

BD: bipolar disorder; SP: schizophrenia; HC: healthy controls; OI: olfactory identification; TMT-A: Trail Making Test-A; DCT: Digit Cancellation Test; TNF-α: tumor necrosis factor-α

*P < 0.05

### Comparison of olfactory function among the three groups

**Fig. 1:**
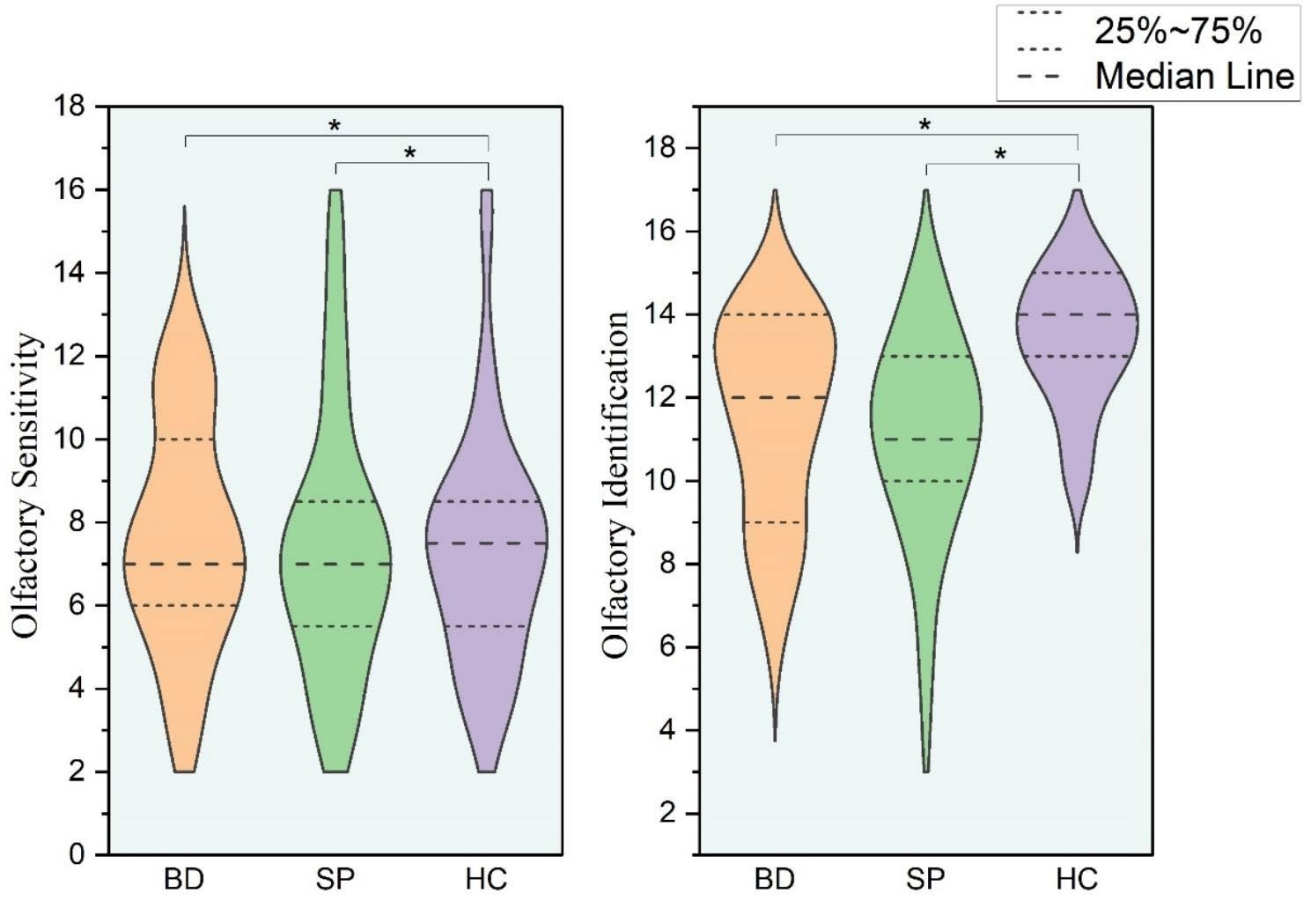
Comparison of olfactory performance among all groups BD: bipolar disorder; SP: schizophrenia; HC: healthy controls *P < 0.05

After the test, the olfactory function data of the three groups did not conform to a normal distribution, and therefore, the non-parametric rank sum test (Kruskal–Wallis *H*-test) was selected for comparisons among the three groups. There was no significant difference in OS among the three groups (*P* > 0.05). As shown in Fig. 1, there was no significant difference in OS between BD or SP patients in remission and HCs. However, OI among the three groups significantly differed (*H* = 25.419, *P* < 0.001). Further pairwise comparison was performed, and the Bonferroni method was used to correct the *P*-value. OI in the BD and SP groups significantly differed from that in the HC group (*P* < 0.001) (Tables 1 and 2; Fig. 1).

Correlation analysis of general data and clinical features showed that OI in the BD group was positively correlated with years of education (*r* = 0.306, *P* = 0.038). In the SP group, OI was negatively correlated with age and PANSS score (*r* = − 0.388, *P* = 0.011; *r* = − 0.537, *P* < 0.001) and positively correlated with years of education and GAF score (*r* = 0.457, *P* = 0.002; *r* = 0.579, *P* < 0.001). In addition, correlation analysis between OS, OI and cognitive function in the BD and SP groups showed that OS in the BD group was positively correlated with DCT II and DCT III (*r* = 0.389, *P* = 0.008; *r* = 0.323, *P* = 0.029), while OS and OI in the SP group were negatively correlated with TMT-A time (*r* = − 0.352, *P* = 0.022; *r* = − 0.587, *P* < 0.001) and positively correlated with DCT III (*r* = 0.329, *P* = 0.033; *r* = 0.350, *P* = 0.023). No significant correlations were found for the other items (*P* > 0.05) (Table 3).

**Table 3:** Correlations between olfactory performance and demographic characteristics, GAF and cognitive function in the BD and SP groups

Variable	BD: OS		BD: OI		SP: OS		SP: OI	
	*r*	P Value	*r*	P Value	*r*	P Value	*r*	P Value
Age	−0.111	0.465	−0.163	0.278	−0.142	0.370	−0.388	0.011*
Duration of illness	−0.112	0.457	−0.140	0.354	0.046	0.770	−0.080	0.614
Education	0.080	0.598	0.306	0.038*	−0.085	0.595	0.457	0.002*
GAF	0.244	0.102	0.127	0.400	0.253	0.106	0.579	< 0.001*
TMT-A time	−0.200	0.183	−0.244	0.102	−0.352	0.022*	−0.587	< 0.001*
DCT scoreI	0.214	0.154	0.096	0.527	0.305	0.050	0.238	0.129
DCT scoreII	0.389	0.008*	0.223	0.137	0.252	0.108	0.294	0.059
DCT score III	0.323	0.029*	0.235	0.116	0.329	0.033*	0.350	0.023*

BD: bipolar disorder; SP: schizophrenia; OS: olfactory sensitivity; OI: olfactory identification; GAF: Global Assessment Function; TMT-A: Trail Making Test-A; DCT: Digit Cancellation Test

*P < 0.05

### Comparison of serum TNF-α among the three groups

Significant differences were found in serum TNF-α level among the groups (*H* = 23.717, *P* < 0.001). Further pair-to-pair comparison and the Bonferroni method showed that serum TNF-α levels in BD and SP patients in remission were significantly lower than those in the HC group. There was no significant difference in serum TNF-α levels between the BD and SP patients (Tables 1 and 2).

There was no significant correlation between olfactory function and serum TNF-α in the BD or SP group (Spearman, *P* > 0.05). Serum TNF-α in the BD group was positively correlated with DCT II (*r* = 0.321, *P* = 0.029). There were no significant correlations for the other items (Table 4).

**Table 4:** Correlations between TNF-α and olfactory performance and cognitive function in the BD and SP groups

Variable	BD group: TNF-α		SP group: TNF-α	
	***r***	**P Value**	***r***	**P Value**
OS	0.155	0.304	0.296	0.057
OI	0.224	0.134	0.075	0.638
TMT-A time	−0.227	0.129	−0.018	0.908
DCT score I	0.104	0.491	0.089	0.576
DCT score II	0.321	0.029*	−0.076	0.633
DCT score III	−0.136	0.369	−0.023	0.887

TNF-α: tumor necrosis factor-α; BD: bipolar disorder; SP: schizophrenia; OS: olfactory sensitivity; OI: olfactory identification; TMT-A: Trail Making Test-A; DCT: Digit Cancellation Test

*P < 0.05

## Discussion

Because of the neuroanatomical overlap between the olfactory system and abnormal brain regions in BD and SP, it is speculated that the olfactory system may be closely related to BD and SP [25]. Indicators, such as OI, can reflect cognitive function [26], while changes in inflammatory cytokines play an important role in cognitive deficits [27]. Because the pathophysiological mechanisms of both diseases may involve mild systemic inflammation/immune dysregulation [17], there may be some relationships between olfactory function, cognitive function and inflammatory cytokines (such as TNF-α) in BD and SP.

### Comparison of olfactory function in each group

Here, we found that, compared with the HC group, OI impairment was present in both BD and SP patients in remission, but there was no significant difference between the two groups. There was no significant difference in OS among all three groups.

Previous studies on BD support our current findings. Li SB et al. [2] found that patients with BD in the remission stage had OI defects, while OS was not significantly different from that in the HC group. Kazour et al. [28] showed that patients with bipolar mania in remission had defects in OI, but had no significant difference in OS compared with HC. The results of some studies differ from our present findings. Hardy et al. [29] found that, compared with the HC group, the OS and OI of patients with BD in the stable stage were not significantly different. The inconsistency may be related to the small sample size of this study.

Some previous studies on SP are in agreement with our current results. Lui et al. [10] and Urban-Kowalczyk et al. [30] both reported that the OI of SP patients in remission was significantly lower than that of HCs. However, Malaspina et al. [11] reported that the OS and OI of SP patients in remission were not significantly different from those in the HC group. The disagreement in findings may be related to differences in factors such as gender, age and disease course.

At present, there are few studies comparing olfactory function between BD and SP patients, and the results have been inconsistent. Li SB et al. [2] reported that OS and OI in SP patients were significantly lower than those in HCs, and OI was the most severely affected among all groups. Cumming et al. [12] showed that the impairment of OI in the SP group was more severe than that in the bipolar group. Kamath et al. [31] compared the olfactory function of SP patients (including SP or schizoaffective disorder, *n* = 64), affective disorder patients (including BD with psychotic symptoms and severe depression with psychotic symptoms, *n* = 25) and HCs (*n* = 98). These investigators showed that the OS and OI of the affective disorder group were not significantly different from those in the HC group, but that OS and OI in the SP group were significantly lower than those in the other two groups. The contrasting results may stem from the fact that the studies did not differentiate among disease subtype or stage at onset. Unfortunately, there are no reports in literature on direct comparison between olfactory function in BD and SP in the remission stage.

### Comparison of cognitive function among the groups and its correlation with olfactory function

Here, patients with BD and SP in remission exhibited functional deficits in attention. In the BD group, OS was positively correlated with DCT II and DCT III, while in the SP group, OS and OI were positively correlated with DCT III. These results show that the higher the degree of cognitive deficits in patients with BD in remission, the lower the OS. Similarly, the higher the degree of cognitive deficit, the worse the OS and OI in patients with SP in remission. Previous studies support this result. Jiménez-López et al. [32] reported that, compared with the HC group, cognitive function in BD and SP patients in remission was worse, while there was no significant difference in cognitive function between the two patient groups.

Hardy et al. [29] reported that performance in the TMT-A in BD patients in remission was worse than that in the HC group, similar to our present findings. Hardy et al. [29] also found that OS in stable BD patients was correlated with clinical symptoms and employment function, but not with cognitive function, inconsistent with the results of the current study. The discrepancy may be related to differences in case group composition and sample size among the studies. In this study, the total number of BD patients was 46, with 25 males, accounting for 54.3% of patients, while in Hardy’s study, the total number of BD patients was 20, with only 5 males, accounting for 25% of patients. Previous studies suggest that gender is related to OS [33]. In addition, differences in evaluation methodology also may contribute to the contrasting results. The SST method was adopted in the current study, while the Smell Threshold Test (STT) was used to detect the OS in Hardy’s study. Lahera et al. [34] reported that OI was significantly related to facial emotion recognition, based on the theory of mind and general cognition. Similar conclusions could not be drawn in this study, possibly due to differences in the disease period and the tools used to assess cognitive function. Some studies suggest that the amygdala, ventromedial prefrontal cortex and orbitofrontal lobe are responsible not only for olfactory processing [8], but also for combining emotion with cognition function in BD patients [35], which seems to underlie the correlation between olfactory and cognitive functions.

Some studies support our results on SP. Malaspina et al. [36] found that reduced OS predicted more severe cognitive deficits. De Nijs et al. [37] showed that OI in SP patients is related to cognitive functions (such as IQ, immediate recall, delayed recall, recognition, attention, processing speed and executive function) and social cognitive functions (such as face recognition, emotion recognition and theory of mind). Nora et al. [38] showed a negative correlation between OI and TMT-A time in SP patients; that is, the better the OI, the stronger the processing speed, attention and cognitive ordering ability, consistent with our present findings. There is a correlation between olfactory function and cognitive function in patients with SP, which may be related to a common neuroanatomical basis. For example, abnormalities in the orbitofrontal region is not only associated with the impairment of olfactory function [39], but also with the impairment of cognitive function [40]. However, a simultaneous comparison of olfactory function and cognitive function between BD and SP patients has not been reported. As the sample size of this study is small, the assessment tools for cognitive function were relatively simple, and the influence of therapeutic drugs cannot be excluded. Future clinical studies with larger group sizes, more cognitive items and rigorous design are needed to verify our current findings.

### The correlation between serum TNF-α and olfactory or cognitive functions in each group

In this study, we found that serum TNF-α was positively correlated with the transferability of attention in patients with BD in remission, contrasting with previous reports. Studies have suggested that serum TNF-α levels in BD patients are negatively correlated with cognitive function [18, 41], and that high levels of serum TNF-α and its receptors produce neurotoxic effects, directly damaging neurons and disrupting neuroplasticity, thereby affecting memory, learning and overall cognitive function. There is imaging evidence that neural structural changes in brain regions responsible for executive function, visual processing, and attention-related activities are associated with TNF-α in BD patients [42]. It has also been reported that there is no correlation between serum TNF-α levels and cognitive function. A recent meta-analysis of BD, SP and depression by Morrens et al. found no significant association between serum TNF-α levels and cognitive function [43]. The inconsistencies among the various studies may be related to the different evaluation tools and indicators of cognitive function employed, and the high heterogeneity of cases. Patients were not divided according to disease type or disease onset stage in the meta-analysis of Morrens et al. In contrast, in the present study, BD and SP patients in remission were grouped separately.

Here, we found no correlation between olfactory function and serum TNF-α in patients with BD and SP in remission. No similar reports have been published previously. Tognetti et al. [44] studied 20 young healthy people to explore whether systemic inflammation leads to olfactory dysfunction. They showed that OI ability was not affected by acute systemic inflammation caused by injection of lipopolysaccharide (LPS), and OI after injection of LPS was unrelated to the level of TNF-α, in line with the present study. We found no correlation between olfactory function and serum TNF-α level in BD or SP patients in remission, which may be related to the complex etiopathogenesis of these diseases, the long course of disease in the patient group in this study, the heterogeneity of the inflammatory response time, and numerous other factors. Therefore, a linear correlation between the two may not be anticipated. In addition, the samples in this study were not large in the case group, and therefore, additional studies are needed to clarify the relationship between olfactory function and serum TNF-α level, and provide a basis for further studies on the etiopathogenetic similarities and differences between BD and SP.

## Conclusions

In this study, olfactory function, serum TNF-α and cognitive function were compared in BD and SP patients in remission. OI impairment was found in BD and SP patients in remission, but there were no significant differences in olfactory function, cognitive function or serum TNF-α between the two groups. These results suggest that OI may be an indicator of BD and SP. Although there was no significant correlation between olfactory function and serum TNF-α level in patients with BD and SP in remission, there was a correlation between cognitive function and olfactory function or serum TNF-α, suggesting that improving olfactory function or alleviating the systemic inflammatory response may be beneficial to the recovery of cognitive function, so as to promote the rehabilitation of patients with SP and BD. Further studies are needed to confirm the feasibility of olfactory function, cognitive function, and serum TNF-α as potential diagnostic and prognostic tools in patients with BD and SP.

There are some limitations in this study. First, the cognitive function assessment method in this study mainly evaluated attention and executive function, but not other functions. In the future, we might consider adding cognitive tests that assess other aspects of cognitive function. Secondly, the olfactory detection method used in this study was relatively subjective. In future studies, we will consider objective methods of assessing olfactory function. Third, the sample size of this study was not large. Fourthly, this study did not exclude the influence of treatment measures or medication on olfactory function, cognitive function or serum TNF-α in patients. More rigorously designed large sample studies should be conducted to exclude the impact of psychotropic medications.

## Data Availability

The datasets used and/or analyzed during the current study are available from the corresponding author on reasonable request.

## Abbreviations

BD: Bipolar disorder
DCT: Digit Cancellation Test
GAF: Global Assessment Function
HAMA: Hamilton Rating Scale for Anxiety
HAMD: Hamilton Rating Scale for Depression
HC: Healthy controls
OI: Olfactory identification
OS: Olfactory sensitivity
PANSS: Positive and Negative Syndrome Scale
SP: Schizophrenia
TMT-A: Trail Making Test-A
TNF-α: Tumor necrosis factor-α
YRMS: Young Mania Rating Scale
